# Efficacy and safety of artemisinin combination therapy (ACT) for non-falciparum malaria: a systematic review

**DOI:** 10.1186/1475-2875-13-463

**Published:** 2014-11-26

**Authors:** Benjamin J Visser, Rosanne W Wieten, Daniëlle Kroon, Ingeborg M Nagel, Sabine Bélard, Michèle van Vugt, Martin P Grobusch

**Affiliations:** Department of Infectious Diseases, Division of Internal Medicine, Center of Tropical Medicine and Travel Medicine, Academic Medical Center, University of Amsterdam, Meibergdreef 9, PO Box 22700, 1100 DE Amsterdam, The Netherlands; Centre de Recherches de Médicales de Lambaréné (CERMEL), Albert Schweitzer Hospital, Lambaréné, Gabon; Institute of Tropical Medicine, University of Tübingen, Tübingen, Germany; Medical Library, Academic Medical Centre, University of Amsterdam, Amsterdam, The Netherlands; Department of Paediatric Pneumology and Immunology, Charité-Universitätsmedizin Berlin, Berlin, Germany

**Keywords:** Artemisinin combination therapy, ACT, Non-falciparum malaria, Plasmodium vivax, Plasmodium ovale, Plasmodium malariae, Plasmodium knowlesi

## Abstract

**Background:**

Artemisinin combination therapy (ACT) is recommended as first-line treatment for uncomplicated *Plasmodium falciparum* malaria, whereas chloroquine is still commonly used for the treatment of non-falciparum species (*Plasmodium vivax*, *Plasmodium ovale* and *Plasmodium malariae*). A more simplified, more uniform treatment approach across all malaria species is worthwhile to be considered both in endemic areas and for malaria as an imported condition alike.

**Methods:**

A PROSPERO-registered systematic review to determine the efficacy and safety of ACT for the treatment of non-falciparum malaria was conducted, following PRISMA guidelines. Without language restrictions, Medline/PubMed, Embase, Cochrane Central Register of Controlled Trials, Web of Science, LILACS, Biosis Previews and the African Index Medicus were searched for studies published up to November 2014.

**Results:**

The literature search identified 986 reports; 40 publications were found eligible for inclusion, all of them on non-falciparum malaria in endemic areas. Most evidence was available for *P. vivax* (n = 35). Five clinical trials in total were identified evaluating ACT for *P. ovale*, *P. malariae* and *Plasmodium knowlesi*. Most ACT presentations have high efficacy against *P. vivax* parasites; artemisinin-based combinations have shorter parasite and fever clearance times compared to chloroquine. ACT is as effective as chloroquine in preventing recurrent parasitaemia before day 28. Artemisinin-based combinations with long half-lives show significantly fewer recurrent parasitaemia up to day 63. The limited evidence available supports both the use of chloroquine and an ACT for *P. ovale* and *P. malariae*. ACT seems to be preferable for optimal treatment of *P. knowlesi*.

**Conclusion:**

ACT is at least equivalent to chloroquine in effectively treating non-falciparum malaria. These findings may facilitate development of simplified protocols for treating all forms of malaria with ACT, including returning travellers. Obtaining comprehensive efficacy and safety data on ACT use for non-falciparum species particularly for *P. ovale*, *P. malariae* and *P. knowlesi* should be a research priority.

**Trial registration:**

CRD42014009103

**Electronic supplementary material:**

The online version of this article (doi:10.1186/1475-2875-13-463) contains supplementary material, which is available to authorized users.

## Background

Malaria remains one of the most important and potentially life-threatening parasitic diseases of humans. Five species of the genus *Plasmodium* are known to cause human malaria infections. The majority of cases are caused by *Plasmodium falciparum*, which can lead to high morbidity and mortality if not treated promptly. Non-falciparum malaria is caused by *Plasmodium vivax*, *Plasmodium ovale* subspecies, *Plasmodium malariae* or *Plasmodium knowlesi*. Data on the epidemiological patterns of the non-falciparum malarias is scarce. In Asia and the Americas, where malaria transmission is generally low and seasonal, *P. falciparum* and *P. vivax* malaria have nearly the same prevalence. *Plasmodium malariae* and *P. ovale* are mainly found in sub-Saharan Africa and comprise <10% of isolates. Currently, non-falciparum malaria constitutes approximately 25% of all annually imported 11,000 cases of malaria in Europe[1–3]. Artemisinin combination therapy (ACT) is recommended as first-line treatment for uncomplicated falciparum malaria[4]. It is considered for traveller’s malaria and *P. knowlesi* in endemic areas, whereas chloroquine (CQ) is still the standard drug for *P. vivax*, *P. malariae* and *P. ovale* in most countries[5].

The different treatment recommendations for *P. falciparum* and non-falciparum malaria may have several drawbacks in clinical practice. Most importantly, misclassification of *Plasmodium* spp. is common especially where non-falciparum infections are involved, due to lack of training in microscopy and variations in morphological characteristics within and between *Plasmodium* spp.[6, 7]. CQ treatment of misclassified *P. falciparum* can have severe or even fatal consequences due to widespread CQ resistance of *P. falciparum*. Despite internal and external quality assessments for parasitological diagnosis, discrepant results between microscopy and polymerase chain reaction (PCR) can still occur even in experienced laboratories[8]. Besides, mixed species infections are common[9] and microscopic diagnosis of mixed-species infections is particularly cumbersome[10, 11]. Rapid diagnostic tests (such as the NOW® ICT MALARIA *P.f*/*P.v*. test based on *P. falciparum* HRP2/aldolase), are not capable to differentiate safely single species *P. falciparum* infection from concurrent *P. malariae* or *P. ovale*[12]. Those tests fail probably because of the small quantities of antigen circulating due to a low parasitaemia in *P. malariae* and *P. ovale*. An evaluation of three rapid diagnostic test (OptiMAL-IT, BinaxNOW® Malaria and Paramax-3) for the detection of *P. knowlesi* infection demonstrated a low sensitivity (OptiMAL-IT 71%, BinaxNOW® 29% and Paramax-3 RDT 40% for fresh *P. knowlesi* samples) and low specificity and a risk of misdiagnosis of *P. falciparum* or *vivax*[13]. Although PCR can unambiguously differentiate between subspecies, it is not used in routine clinical care.

Secondly, *P. vivax* resistance to CQ is emerging in parts of sub-Saharan Africa and South East Asia[14]. A recent meta-analysis to establish the global extent of chloroquine resistance showed chloroquine resistance in 58 (53%) of 113 assessable study sites, spread across most countries that are endemic for *P. vivax*[15]. The World Health Organization (WHO) recommends the use of ACT also for the treatment of *P. vivax* in affected areas[16]. So far, resistance of *P. ovale* and *P. malariae* to CQ has been reported only once from Indonesia[17].

Finally, intake of CQ is unpopular in many African descendants due to frequently experienced side effects, e.g. pruritus[8] due to CQ’s affinity to melanocytes[18]. Artemisinin derivatives are generally well tolerated, and the safety profile of ACT may be by-and-large determined by the partner drug[19]. The clinical impact of non-falciparum malaria infection seems to be more important as it was considered the last three decades. A systematic review and meta-analysis showed *P. vivax* as a major cause of severe malaria and a comparable incidence of severe malaria between *P. vivax* and *P. falciparum* was found in infants, children and adults[20].

Hence, there might be a compelling rationale to treat all (including returning travellers) diagnosed with malaria with an ACT irrespective of malaria species[21]. More or less identical first-line treatments based on ACT for all malaria species would not only simplify treatment schemes and avoid ineffective CQ treatment of unrecognized *P. falciparum* infection, but also offer logistical benefits in terms of pharmacological management, whilst the need to eliminate *P.vivax*/*P.ovale* hypnozoites outside endemic areas has to be accounted for. Moreover, using a combination treatment will less likely result in artemisinin resistance development. However, certainty about the efficacy and safety of ACT for non-falciparum malaria and mixed malaria infections is needed before translating ACT treatment for all malaria infections into practice. This review aims at describing the efficacy and safety of ACT for non-falciparum malaria.

The overall objective of the review was to summarize available data on efficacy and safety of artemisinin combination therapy (ACT) for the treatment of non-falciparum malaria. Primary endpoint was to describe the cure rate at day 28; secondary endpoints were to describe cure rates at days 0–3, 7, 14, 21, 28, 42, 56 and 63, fever clearance time (FCT), parasite clearance time (PCT) and adverse events profiles[22].

## Methods

This review was conducted in June 2014. The last search was conducted on 16 November 2014. Objectives and inclusion criteria were specified in advance and documented in a protocol. Recommendations made by the Preferred Reporting Items for Systematic Reviews and Meta-Analyses (PRISMA) group were followed[23]. This review was registered in advance in PROSPERO (International prospective register of systematic reviews). Registration number: CRD42014009103[24]. The full methods section is described in Additional file1. For the search strategy and selection criteria, see Additional file2.

## Results

**Figure 1:**
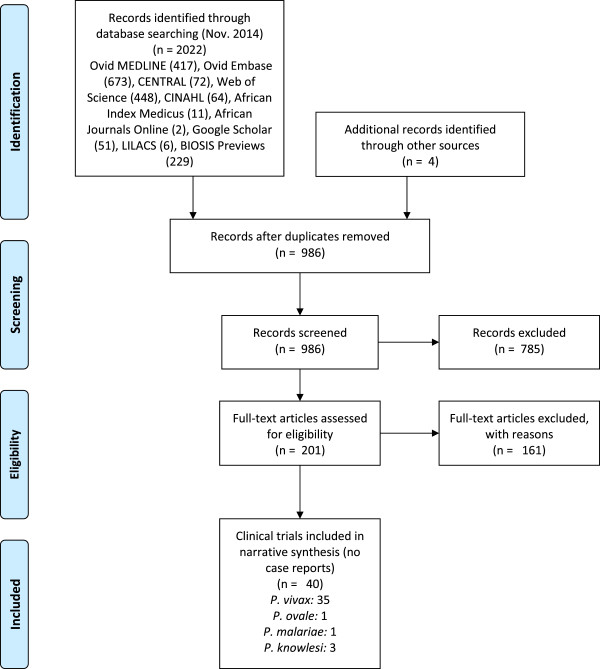
**PRISMA flow diagram.** *From*: Moher D, Liberati A, Tetzlaff J, Altman DG, The PRISMA Group (2009). Preferred Reporting Items for Systematic Reviews and Meta-Analyses: The PRISMA Statement. *PLoS Med* 6: e1000097.

The initial search yielded 2,022 records of which 986 remained after removal of duplicates (see PRISMA flow diagram, Figure 1). Forty records met the inclusion criteria. Of these, n = 35 for *P. vivax* malaria and n = 5 altogether for *P. malariae*, *P. ovale* or *P. knowlesi* contained the necessary data for the qualitative synthesis. The full text was retrievable for all included records except two[25, 26]. No study for the treatment of travellers with non-falciparum malaria with ACT in non-endemic high-income countries was identified.

### Treatment of *Plasmodium vivax*malaria

The epidemiology, clinical presentation and diagnosis of *P. vivax* are not in the scope of this review and have been covered elsewhere[27].

### ACT *versus*CQ

Fifteen randomized clinical trials (Table 1) compared ACT with CQ monotherapy (with or without primaquine (PQ); the studied artemisinin-based combinations were artemether-lumefantrine (AL)[28–32], dihydroartemisinin-piperaquine (DHA + PP)[33–35], artesunate plus sulphadoxine-pyrimethamine (AS + SP)[36–38], artesunate-pyronaridine (AS + PY)[39], artemisinin-naphtoquine (AN)[40], or artesunate (AS)[41] for severe *P. vivax* malaria[42]. ACT clears *P. vivax* parasitaemia significantly faster than CQ[43] (Table 1). In addition, ACT also appears to reduce fever clearance time faster than CQ (Table 1). Overall, in regions where there is no CQ resistant *P. vivax* malaria, ACT is as effective as CQ at preventing recurrent parasitaemia up to day 28[31, 34–36, 39, 44]. Five trials[28, 34–36, 44] followed up beyond day 28, but in only one, primaquine was administered to achieve radical cure[39]. It was demonstrated that AS + PY followed by PQ reduced the risk of recrudescent parasitaemia between day 28 and 42[39]. The longest follow-up was 63 days in a study testing DHA + PP, where DHA + PP treated patients had a 79.1% cumulative risk of recurrence with *P. vivax* at nine weeks (95% CI 73.5%-84.8%) in patients treated with CQ, compared with 54.9% (95% CI, 48.2%-61.6%) in those receiving DHA + PP[34]. In all trials comparing ACT with CQ, serious adverse events were rare. ACT has also been investigated in non-randomized clinical trials with AL[32, 45–47] and injectable artesunate (for severe *P. vivax* malaria)[25, 26, 41, 42, 48, 49], all showing relative short parasite and fever clearance times and excellent day 28 cure rates.

**Table 1:** **Clinical trials of varying design that specifically report on the effectiveness of an artemisinin derivate combined with a blood schizonticide for the treatment of** ***Plasmodium vivax***, ***P. malariae***, ***P. ovale*** **or** ***P. knowlesi*** **malaria**

First author, year of publication	Country	Study design	Drug (days)	N	PCT (h)	FCT (h)	Cure rate									Statistical significance
							Day 1	Day 2	Day 3	Day 7	Day 14	Day 28	Day 42	Day 56	Day 63	
***Plasmodium vivax***																
*Lon et al. 2014*[50]^*α*^	Cambodia	RCT, open label	DHA + PP (2)	32	8 h	16 h	NR	NR	NR	NR	NR	NR	91%	NR	NR	Day 42: P = 0.62
			DHA + PP (3)	28	8 h	16 h	NR	NR	NR	NR	NR	NR	96%	NR	NR	
*Liu et al.2013*[44]^*α*^	China	RCT, open label	AN (3)	127	26.0 h	26.9 h	80.3%	80.3%	100%	NR	NR	100%	98.4%	NR	NR	Day 42: p = 0.4496
			CQ + PQ (3 + 8)	128	37.4 h	39.8 h	40.6%	54.7%	100%	NR	NR	100%	96.1%	NR	NR	
*Leang et al. 2013*[33]^*β*^	Cambodia	CCT	DHA + PP	168	NR	NR	85.5%	100%	100%	NR	NR	100%	NR	NR	NR	NR
			CQ	211	NR	NR	18.9%	83.9%	98.2%	NR	NR	90.5%	NR	NR	NR	
*Pasaribu et al. 2013*[51]^*α*^	Indonesia	RCT, open label	AAQ + PQ (3 + 14)	167	<48 h	NR	NR	100%	NR	NR	NR	NR	91%	NR	NR	Day 42: p = 0.51
			DHA + PP + PQ (3 + 14)	164	<48 h	NR	NR	98.1%	NR	NR	NR	NR	94%	NR	NR	
*Sutanto et al. 2013*[52]^*α*^	Indonesia	RCT, open label	AS (6)	41	<72 h	NR	NR	NR	100%	100%	100%	39,0%	NR	NR	NR	NR
			DHA + PP + PQ (3 + 14)	39	<72 h	NR	NR	NR	94.9%	100%	100%	100%	NR	NR	NR	
			QN + PQ (7 + 14)	36	<72 h	NR	NR	NR	97.2%	100%	100%	100%	NR	NR	NR	
*Hwang et al. 2013*[28]^*α*^	Ethiopia	RCT, open label	AL (3)	114	NR	NR	NR	100%	100%	NR	NR	91.1%	58.8%	NR	NR	Day 2: p = 0.006
			CQ (3)	107	NR	NR	NR	94%	99,1%	NR	NR	97,2%	68.4%	NR	NR	Day 28: p = 0.003
*Senn et al*. 2013[45]^*α*^	Papua New Guinea	CT	AL (NR)	594	NR	NR	NR	NR	NR	99.8%	NR	97.8%	88%	NR	NR	NR
*Mohapatra et al. 2013*[41]*NR*	India	CT	AS (7)	60	48.2 h	47.8 h	NR	NR	NR	NR	NR	96.6%	NR	NR	NR	NR
*Barber et al. 2013*[46]*NA*	Malaysian Borneo	CT	AL + PQ (+AS)	43	48 h	24 h	NR	NR	NR	NR	NR	NR	NR	NR	NR	NR
*Abdallah et al*. 2012[47]^*α*^	Sudan	CT	AL + PQ (3 + 14)	38	NR	NR	94.7%	100%	NR	NR	NR	100%	NR	NR	NR	NR
*Eibach et al. 2012*[32]^*β*^	Guyana	CT	AL + PQ (3 + 14)	61	NR	NR	100%	NR	NR	NR	100%	97%	NR	NR	NR	NR
*Phyo et al. 2011*[34]^*α*^	Thailand	RCT	DHA + PP (3)	248	NR	NR	63,7%	97,5%	100%	NR	NR	95,9%	NR	NR	45.1%	Day 1: p < 0.001
			CQ(3)	244	NR	NR	21,1%	84,1%	97.9%	NR	NR	90.2%	NR	NR	20.9%	Day 2: p < 0.001
																Day 28: p = 0.032
																Day 63: p < 0.001
*Poravuth et al. 2011*[39]^*β*^	Cambodia, Thailand, India and Indonesia	RCT, Phase III	AS + PY (3) + PQ	228	23.0 h	15,9 h	NR	NR	NR	NR	99.5%	NR	NR	NR	NR	PCT: p < 0.0001
																FCT: p = 0.0017
			CQ (3)	228	32.0 h	23,8 h	NR	NR	NR	NR	100%	NR	NR	NR	NR	
																Difference (95% CI)
																Day 14: -0.5 (-2,6-1,4)
*Yohannes et al. 2011*[29]^*α*^	Ethiopia	CCT	AL (3)	88	NR	NR	NR	NR	NR	NR	NR	81.0%	NR	NR	NR	Day 28: p = 0.0145
			CQ (3)	71	NR	NR	NR	NR	NR	NR	NR	92.5%	NR	NR	NR	
*Awab et al. 2010*[35]^*α*^	Afghanistan	RCT, open label	DHA + PP (3)	264	NR	NR	91.3%	97.8%	NR	NR	NR	100%	NR	97.2%	NR	Day 1: p < 0.001
			CQ (3)	266	NR	NR	78.9%	97.0%	99,2%	NR	NR	100%	NR	91.1%	NR	Day 2: p = 0.59
																Day 56: p = 0.003
*PiDan et al. 2009*[25] (*abstract only*)	China	CT	AS (5)	30	16.3 h	38.3 h	NR	NR	NR	NR	NR	NR	NR	NR	NR	Cure rate: 53,3% (AS) vs. 56.7% (AH). Day: NR
			AH (3)	30	23.8 h	38.5 h										
*Krudsood et al. 2008*[53]^*β*^	Thailand	RCT	(1)AS + PQ (5 + 5)	68	34.9 h	16.8 h	NR	NR	NR	NR	NR	85%	NR	NR	NR	Group 1 ≠ group 4 and group 5 (p = 0.004 and 0.003). Group 2 ≠ group 4 and group 5 (p = 0.015 and 0.014)
			(2) AS + PQ (5 + 7)	69	36.0 h	20.0 h	NR	NR	NR	NR	NR	89%	NR	NR	NR	
			(3) AS + PQ (5 + 9)	66	35.7 h	14.5 h	NR	NR	NR	NR	NR	96%	NR	NR	NR	
			(4) AS + PQ (5 + 11)	64	34.2 h	13.8 h	NR	NR	NR	NR	NR	100%	NR	NR	NR	
			(5) AS + PQ (5 + 14)	66	37.2 h	19.6 h	NR	NR	NR	NR	NR	100%	NR	NR	NR	
			(6) AS + PQ (5 + 7) double dose PQ	66	36.8 h	21.4 h	NR	NR	NR	NR	NR	96%	NR	NR	NR	
*Karunajeewa et al. 2008*[38]^*β*^	Papua New Guinea	RCT	AL (3)	39	1.4d	2.1d	NR	NR	NR	NR	NR	55.5%	45.5%	NR	NR	PCT: ACTs vs.
																CQ + SP: p = 0.05
			AS + SP (3)	51	1.1d	2.1d	NR	NR	NR	NR	NR	63.8%	51.3%	NR	NR	FCT: p > 0.05
			DHA + PP (3)	44	1.2d	1.9d	NR	NR	NR	NR	NR	84.2%	72.2%	NR	NR	
			CQ + SP (3)	61	3.1d	2.3d	NR	NR	NR	NR	NR	58.8%	44.7%	NR	NR	
*Dao et al. 2007*[42]^*β*^	Vietnam	CT	AS + PQ (2 + 7)	28	14.2 h	18.6 h	NR	NR	NR	NR	NR	96.2%	NR	NR	NR	NR
*Hasugian et al. 2007*[54]^*α*^	Papua, Indonesia	RCT	AAQ + PQ (3 + 14)	75	<48 h	<48 h	NR	NR	NR	NR	NR	NR	52%	NR	NR	Day 42: p < 0.001
			DHA + PP + PQ (3 + 14)	74	<48 h	<48 h	NR	NR	NR	NR	NR	NR	84%	NR	NR	
*Ratcliff et al. 2007*[55]^*α*^	Papua, Indonesia	RCT	AL (3)	141	NR	NR	NR	NR	NR	NR	NR	NR	43%	NR	NR	p < 0.0001
			DHA + PP (3)	147	NR	NR	NR	NR	NR	NR	NR	NR	86%	NR	NR	
*Krudsood et al. 2007*[31]^*α*^	Thailand	RCT	AL + PQ (3 + 14)	47	41.6 h	21.8 h	NR	NR	NR	NR	NR	97.4%	NR	NR	NR	PCT: p < 0.001
																FCT: p = 0.12
			CQ + PQ (3 + 14)	51	55.8 h	25.3 h	NR	NR	NR	NR	NR	100%	NR	NR	NR	
*Kolaczinski et al. 2007*[36]^*α*^	Afghanistan	RCT	AS + SP (3 + 1)	94	NR	NR	94.4%	100%	100%	100%	100%	99%	76%	NR	NR	Difference (95% CI)
																Day 28: 3.3% (-2.3—9.8)
			CQ (3)	96	NR	NR	72.2%	97.8%	100%	100%	100%	96%	54%	NR	NR	
																Day 42: 21.1% (7.2—34.0)
*Yoda et al. 2005*[26] (*abstract only*)	Thailand	CT	AS (5) + PQ (14)	42	36.7 h	14.6 h	NR	NR	NR	NR	NR	95.2%	NR	NR	NR	NR
*Hamedi et al. 2004*[48]^*α*^	Thailand	CT	AS + PQ (5 + 14)	42	36.7 h	14.6 h	NR	NR	NR	NR	100%	95.2%	NR	NR	NR	NR
*Karunajeewa et al. 2003*[49]^*α*^	Papua New Guinea	CT	AS (1)	5	NR	NR	80%	NR	NR	NR	NR	NR	NR	NR	NR	NR
*Silachamroon et al. 2003*[56]^*α*^	Thailand	RCT	AS (5)	157	39.3 h	21.2 h	NR	NR	NR	NR	NR	47.8%	NR	NR	NR	Day 28: p < 0.0001. AS + PQ (both) vs. AS(both)
			AS (7)	159	39.6 h	21.7 h	NR	NR	NR	NR	NR	52.2%	NR	NR	NR	
			AS + PQ (5 + 14)	142	37.7 h	23.6 h	NR	NR	NR	NR	NR	100%	NR	NR	NR	
			AS + PQ (7 + 14)	157	38.8 h	21.6 h	NR	NR	NR	NR	NR	100%	NR	NR	NR	
*Da Silva Rdo et al. 2003*[57]^*β*^	Brazil	CCT	(a) AS + PQ (1 + 7)	30	NR	NR	NR	96.7%	NR	NR	NR	NR	NR	NR	NR	PCT: AS faster than CQ (p < 0.01)
			(b) AS + PQ (1 + 7)	30	NR	NR	NR	100%	NR	NR	NR	NR	NR	NR	NR	
			(c) AS + PQ (1 + 7)	30	NR	NR	NR	100%	NR	NR	NR	NR	NR	NR	NR	Cure rate: 92.3% (group A + B + C + G) vs. 80.2% (group D + E + F + H) P = 0.0372.
			(d) AS + PQ (1 + 5)	30	NR	NR	NR	100%	NR	NR	NR	NR	NR	NR	NR	
			(e) AS + PQ (1 + 5)	26	NR	NR	NR	96.2%	NR	NR	NR	NR	NR	NR	NR	
			(f) AS + PQ (1 + 5)	28	NR	NR	NR	100%	NR	NR	NR	NR	NR	NR	NR	
			(g) CQ + PQ (1 + 7)	30	NR	NR	NR	76.7%	NR	NR	NR	NR	NR	NR	NR	
			(h) CQ + PQ (1 + 5)	30	NR	NR	NR	60.0%	NR	NR	NR	NR	NR	NR	NR	
*Tjitra et al. 2002*[37]^*α*^	Papua, Indonesia	CCT	AS + SP (3 + 1)	22	1.1d	1.4d	NR	NR	NR	100%	89.5%	NR	NR	NR	NR	CQ vs. CQ + SP p = 0.046
			CQ + SP(3 + 1)	6	NR	NR	NR	NR	NR	NR	67%	NR	NR	NR	NR	
			CQ (3)	9	NR	NR	NR	NR	NR	NR	11%	NR	NR	NR	NR	
*Phan et al*. 2002[58]^*α*^	Vietnam	RCT	AM (3)	113	24 h	16 h	NR	NR	NR	NR	77%	NR	NR	NR	NR	Day 28: p = 0.3
			CQ (3)	113	24 h	16 h	NR	NR	NR	NR	83%	NR	NR	NR	NR	
*Pukrittayakamee et al*. 2000[59]^*α*^	Thailand	RCT	AS (5)	20	38 h	14 h	NR	NR	NR	NR	36.8%	NR	NR	NR	NR	PCT:
			AH (5)	20	50 h	17 h	NR	NR	NR	NR	47.0%	NR	NR	NR	NR	CQ + PQ vs. AS p < 0.005
			PQ (14)	30	93 h	28 h	NR	NR	NR	NR	76.9%	NR	NR	NR	NR	CQ + PQ vs. QN, PQ and SP p < 0.001
			SP (1)	12	114 h	58 h	NR	NR	NR	NR	20%	NR	NR	NR	NR	CQ + PQ and MQ vs. others P < 0.017
			CQ (3)	30	65 h	31 h	NR	NR	NR	NR	71.4%	NR	NR	NR	NR	
			QN (7)	22	98 h	31 h	NR	NR	NR	NR	35.3%	NR	NR	NR	NR	
			MQ (1)	20	76 h	21 h	NR	NR	NR	NR	100%	NR	NR	NR	NR	
			HT (1)	23	85 h	35 h	NR	NR	NR	NR	35.3%	NR	NR	NR	NR	
			CQ + PQ (3 + 14)	30	65 h	30 h	NR	NR	NR	NR	100%	NR	NR	NR	NR	
*Li et al*., *1999*[30]*NR*	China	NR	AL (3) (higher dose)	36	33.5 h	22.3 h	NR	NR	NR	NR	NR	NR	NR	NR	NR	PCT: p < 0.01
																FCT: p > 0.05
			AL (3) (lower dose)	41	30.5 h	22.3 h	NR	NR	NR	NR	NR	NR	NR	NR	NR	Relapse rates Month 9: 84.9%, 78.8% and 22.9%
			CQ + PQ (NR)	55	44.9 h	25.0 h	NR	NR	NR	NR	NR	NR	NR	NR	NR	
*Wilairatana et al*., *1999*[60]^*α*^	Thailand	CCT	SP (1)	23	123.1 h	49.5 h	NR	NR	NR	NR	NR	40%	NR	NR	NR	AS + PQ vs. others P < 0.001
			SP + PQ (1 + 14)	23	96.8 h	43.2 h	NR	NR	NR	NR	NR	100%	NR	NR	NR	
			PQ (14)	23	85.1 h	37.7 h	NR	NR	NR	NR	NR	100%	NR	NR	NR	
			AS + PQ (3 + 14)	23	41.1 h	16.4 h	NR	NR	NR	NR	NR	100%	NR	NR	NR	
***P. ovale***																
*Same*-*Ekobo et al*., *1999*[61]*NR*	Cameroon	CT	AS (NR)	30	38.8 h	36.6 h	NR	NR	NR	NR	NR	NR	NR	NR	NR	
***Plasmodium malariae***																
*Borrmann et al. 2002*[62]^*α*^	Gabon	CCT	AS (3)	35	NR	NR	NR	NR	NR	100%	NR	NR	NR	83%	NR	Day 7: p < 0.0001
			Placebo (3)	23	NR	NR	NR	NR	NR	0%	NR	NR	NR	0%	NR	Day 56: p < 0.0001
**Mixed**, ***Plasmodium ovale and*** **/or** ***Plasmodium malariae and***/***or Plasmodium falciparum***																
*Mombo*-*ngoma et al. 2012*[8]^*α*^	Gabon	CT	AL (3)	38	24 h	NR	82%	100%	NR	NR	NR	100%	NR	NR	NR	
**Mixed infections**: ***Plasmodium falciparum*** **and** ***Plasmodium vivax***																
*Tjitra et al. 2012*[63]^*α*^	Indonesia	RCT	AN (1)	401	13.3 h	28.0 h	NR	NR	NR	NR	NR	NR	96.3%	NR	NR	153 (38%) *P.f*., *158* (*39%*) *P.v*., *90* (*22%*)*P.f*./*P.v*.
			DHP (3)		11.3 h	25.5 h							97.3%			
													(AM)			
*Smithuis et al. 2010*[9]	Myanmar (Burma)	RCT, open label	AAQ	155	NR	NR	NR	NR	NR	NR	NR	92%	NR	NR	90.6%	129 (16%) mixed infections *P.f*. and *P.v*.
			AL	162								99.4%			98.6%	
			AS-MQ (fixed)	169								100%			100%	
			AS-MQ (loose)	161								99.4%			98.7%	
			DHP	161								100%			98.7%	
*Asley et al. 2005*[64]	Thailand	RCT	DHP (3)	164	<48 h	<48 h	NR	NR	NR	NR	NR	NR	NR	NR	100%	45 of 499 patients (9%) with *P.v*. > Results for any malaria.
			DHP (1)	169	<48 h	<48 h									99.4%	
			MQ-AS	166	<48 h	<48 h									95.7%	
***Plasmodium knowlesi***																
*Barber et al. 2011*[65]	Malaysian Borneo	CT	AL	109	48 h	24 h	NR	NR	NR	NR	NR	NR	1 patient with recurrent *P.k*. day 42	NR	NR	Total 130 *P.k*. patients. 23 (18%) of *P.k*. patient with follow up 26–41 days, no recurrences.
			AS-MQ	10												
			(i.v. AS for SM)	36												
*William et al. 2011*[66]	Malaysian Borneo	CT	AL	8	24 h	NR	NR	NR	NR	NR	NR	NR	NR	NR	NR	NR
			AS (for SM)	6	48 h											

Case-series and case-reports are not depicted.

^α^Survival analysis: preferred for statistical analysis of data on drug efficacy. The advantage of survival analysis is that it takes into account data on patients who were lost to follow-up or withdrawn from the study, in particular patients with reinfection.

^β^Per-protocol analysis: in this method, all patients who cannot be evaluated (i.e. those withdrawn, lost to follow-up or reinfected after PCR correction) are removed from the denominator.

### AS + SP

Only one artemisinin combination, namely AS + SP, seems unsuitable for the treatment of vivax malaria. Although one non-inferiority trial[36], from Afghanistan showed good efficacy (day 28 cure rate AS + SP 99% *vs* CQ 96%), it seems that *P. vivax* has developed resistance to pyrimethamine faster than *P. falciparum* did[67]. Consequently, AS + SP may not be effective overall against *P. vivax* in many regions.

### ACT *versus*CQ + SP

Two trials investigated an ACT versus CQ + SP; one study compared AS + SP with CQ + SP[37], and one trial compared three artemisinin-based combinations (DHA + PP, AL and AS + SP) with CQ + SP[38]. High recurrence rates were noted, due to high levels of CQ and/or SP resistance at the study site. The best form of ACT in this trial was DHA + PP, with a day 28 cure rate of 84.2%, significantly better than CQ + SP[38]. Only one trial evaluated the efficacy of ACT compared to quinine[52]. The efficacy of primaquine (PQ) against relapse was 92% (95% CI 81%-96%) for quinine plus PQ and 98% (95% CI 91%-99%) for DHA + PP plus PQ.

### ACT *versus*ACT

Six studies[38, 50–52, 54, 55, 64] evaluated different artemisinin-based combinations. Treatment efficacy with DHA + PP was superior to AL, with higher cure rates at day 28[38, 55]. DHA + PP was also more effective compared to AS + AQ[54] and AS + SP[38] while there was no difference to AS + MQ[64] in preventing recurrent parasitaemia. Thus, most evidence is available on the efficacy and safety of DHA + PP for the treatment of *P. vivax*[28, 33, 34, 38, 50–52, 54, 55, 68]. As depicted in Table 1, no evidence was found on superiority of one regimen over others regarding the parasite and fever clearance time.

An RCT evaluating a two versus three-day regimen of DHA + PP (total dose 360 mg DHA and 2880 mg PP) for uncomplicated malaria (75% *P. vivax*, 6,3% mixed) showed no difference in efficacy for treatment of *P. vivax* or all-species malaria before day 42[50]. Although piperaquine is generally well tolerated with few side effects, there was a safety signal suggested by the degree of QT interval prolongation. The study reported a mean QT prolongation of 20–30 ms between pre-dose and trough drug piperaquine levels, and roughly 18.5% of the 80 subjects dosed had a grade I cardiac adverse event due to QT prolongation during the 3 day period following dosing. There were no differences in adverse cardiac events between the two groups. More recently, a randomized, double-blind, placebo-controlled clinical trial of a compressed two-day regimen of DHA + PP for malaria protective efficacy was halted after 6 weeks (69 of 231 participants enrolled) for concern over prolonged corrected QT interval[69]. Participants received two consecutive daily doses (total dose 360 mg DHA and 2880 mg PP) within 30 min to 3 hour of a meal. The data was reviewed (unblinded) by the data safety monitoring board and they revealed a mean QTcF prolongation of 46 ms over placebo at the maximum concentration of drug in serum (C_max_) on day 2. The authors concluded that a compressed 2-day regimen is best avoided until the clinical relevance of these findings are more thoroughly evaluated.

Evidence from trials evaluating ACT for non-falciparum shows good efficacy of various artemisinin-based combinations for mixed species infection[9, 63]. In an RCT in Myanmar (Burma) 129 (16%) of patients had a mixed species infection, and all patients with mixed infections responded to ACT treatment[9]. Fewer cases of *P. vivax* were identified in those who received artesunate-mefloquine than in patients who received artesunate-amodiaquine, artemether-lumefantrine, or the loose tablet regimen of artesunate-mefloquine[9].

### Treatment of ovale and malariae malaria

Epidemiology of *P. ovale* and *P. malariae*, clinical presentation and diagnosis are described in a recent review[70] and briefly in Additional file3. Only three trials evaluating an artemisinin derivative for *P. ovale* or *P. malariae* could be identified[8, 61, 62]. A clinical study evaluated the efficacy and tolerability of a three-day course of artesunate monotherapy for the clearance of asymptomatic *P. malariae* infections in Gabon[62]. Patients were randomized and received artesunate (n = 63) or placebo (n = 45). Most were mixed infections with *P. falciparum* and 18 were asymptomatic *P. malariae* mono-infections. On day 7, the cure rate was 100% (95% CI 91-100%) for *P. malariae* patients treated with artesunate versus no cure (95% CI 0-15%) in the placebo group. Treatment with artesunate led to a parasitological cure rate of 83% by day 56 in this semi-immune population. However, there may have been possible re-infections during the 56-day follow-up period. The true cure rate is likely to be underestimated as these results were not corrected for by PCR. In another clinical trial, artesunate was 100% effective for the treatment of *P. ovale* infections, with a PCT and FCT of < 48 hours[61]. Treatment was well tolerated and serious adverse events were not reported. Another study in Gabon, a prospective non-randomized trial evaluated the safety and efficacy of an ACT (AL) for non-falciparum malaria[8]. Most patients in this study had a mixed infection (n = 32) and 7 patients presented with a non-falciparum mono-infection. The median parasite clearance time was 36 hours, median FCT 8 hours, and all patients had an adequate clinical and parasitological response at day 28 (95% CI 91-100%). No serious adverse events were recorded. The limitations of this study were its non-randomized design, short follow-up period of 28 days (likely to miss any relapse of *P. ovale*) and no anti-relapse therapy with primaquine administered for *P. ovale*. One other case report is available, which reported good efficacy of intravenous artesunate for mixed *P. falciparum*/*P. malariae* infection[71]. This stands in contrast to a case report from Italy, in which they described a mixed *P. falciparum*/*P. malariae* infection in a HIV-infected male and ineffectiveness of artemether/lumefantrine to clear the infection of *P. malariae*[72]. This could be explained by the ineffectiveness of AL against the long-lasting schizogonic pre-erythrocytic phase of *P. malariae* or a natural induced drug resistance of the parasite, which have never been published before in quartan malaria. However, single case reports leave open questions, and hypotheses on lack of ACT efficacy against *P. malariae* remain to be somewhat weak.

### Treatment of *Plasmodium knowlesi*malaria

A detailed overview on the history, clinical symptoms and diagnosis of *P. knowlesi* malaria is provided in a recent review[73]. Because experience is limited to low case numbers, and as there are no high-quality clinical studies with adequate patient numbers for understandable reasons, current WHO Malaria Treatment Guidelines (2010)[16] do not provide official treatment recommendations yet for uncomplicated knowlesi malaria. For severe knowlesi malaria, WHO recommends intravenous artesunate[74] in their practical handbook ‘Management of severe malaria (2013). Taking into account the biology of the parasite and the resulting clinical features with possible rapid progress to severe disease, ACT appear as a natural choice for first-line treatment. Consequently, hospital guidelines in endemic areas currently recommend an oral ACT (AL) for non-severe *P. knowlesi* malaria, and intravenous artesunate for severe knowlesi malaria[46]. A recent study from Barber and colleagues confirmed the safety and efficacy of AL (n = 109) or artesunate-mefloquine (n = 10) for uncomplicated *P. knowlesi* infections in humans[46]. The mean parasite clearance time (not separated for *P. vivax* or *P. knowlesi*), was two days, with 55/119 (42%) of *P. knowlesi* infected patients being smear-negative by day 1 (P = 0.032). The median fever clearance time was one day in *P. knowlesi* compared to two days for *P. falciparum* malaria. Twenty-three (18%) *P. knowlesi* patients were followed up during days 26–41, with no recrudescence identified. For severe knowlesi malaria, intravenous artesunate was successfully used. In this study, >80% (15 of 18) of the patients with a *P. knowlesi* parasitaemia >100,000 parasites/μL met other severity criteria, supporting the use of this cut-off value for mandating intravenous artesunate. William and colleagues demonstrated excellent efficacy of AL (n = 8) for uncomplicated knowlesi malaria in a retrospective case review study[66]. The median parasite clearance time was significantly shorter for patients receiving AL (one day; range 0–3) and for those receiving CQ (2.5 days; range 1–3) or quinine (2.5 days; range 1–3) (p = 0.01)[66]. Of the 22 patients with severe *P. knowlesi* malaria, 16 were treated with intravenous quinine, six received intravenous artesunate. The median PCT was significantly shorter with artesunate (2 days; range 1–3) than with quinine (4 days; range 2–7) (p = 0.02). Six patients died, of which 5 had been administered quinine (median severity criteria 2; case-fatality rate 31%), and 1 was given artesunate (median severity criteria 2.5; case-fatality rate 16.6%). Only a few others report on the treatment of *P. knowlesi* with ACT. A patient from Pahang Province (Malaysia) was unsuccessfully treated with mefloquine, but recovered with intravenous quinine in combination with artemether-lumefantrine[75]. Another case report describes a case of *P. knowlesi* infection in a traveller from Thailand in Germany. Parenteral treatment was started (2.4 mg/kg) and the patient was switched to oral AL the next day, on which she fully recovered[76].

## Discussion

Evidence for the efficacy of ACTs for *P. falciparum* malaria is extensive, and rapidly growing for *P. vivax* malaria. Clinical trials of ACT for *P. malariae*, *P. ovale* and *P. knowlesi* are much scarcer and limited to non-randomized studies, case-series or case-reports.

### *Plasmodium vivax*malaria

Several artemisinin-based combinations yield high efficacies against *P. vivax* parasites, both sensitive and resistant to CQ. Studies identified in this review showed good efficacy of ACT against the blood stages of *P. vivax*, with quicker parasite and fever clearance times compared to CQ monotherapy. Countries with CQ-resistant *P. vivax* are already implementing a unified ACT-based treatment policy[77, 78]. Increasing resistance of chloroquine against *P. vivax* is, after the first report in 1989 from Papua New Guinea[79], reported in Indonesia[80–83], Myanmar[84], Papua New Guinea[85], Vietnam[86], Turkey[87, 88], Colombia[89], Brazil[90], Brazilian Amazon[91] and Ethiopia[29, 92–94]. A recent systematic review and meta-analysis reviewed *P. vivax* malaria treatment efficacy studies to establish the global extent of chloroquine resistance[15]. Although there was substantial heterogeneity of study design and analysis between the 129 eligible clinical studies (21,694 patients) chloroquine resistance was present in 58 (53%) of 113 assessable study sites, spread across most countries that are endemic for *P. vivax*. Also, within countries close to elimination of malaria, an increasing proportion of *P. vivax* malaria is imported and, outside sub-Saharan Africa, the proportions of all cases caused by *P. vivax* are rising[95]. In travellers returning from supposedly CQ-sensitive *P. vivax* to non-endemic areas, the best treatment remains to be elucidated; in these cases, the economic, malariometric, and operational costs of ACT *versus* CQ need to be weighted by the clinician. Different regimens are still acceptable if diagnostic tests reliably differentiate CQ-resistant *P. falciparum* from *P. vivax*. However, this is even in experienced laboratories not always the case. Moreover, the paradigm among infectious diseases experts that bacterial infections should be treated as “narrow as possible” does not apply to treating malarial infections. Monotherapy for malaria will only increase the likelihood of resistance, in contrast with narrowing down antibiotic therapy resulting in lower levels of bacterial resistance[96].

A position paper (2012) from the European Society for Clinical Microbiology and Infectious Diseases, summarized main issues regarding the management of imported malaria[97]. For the treatment of non-falciparum malaria, they recommended chloroquine as the drug of choice and ACT a pragmatic alternative (especially in case of chloroquine resistance). Currently, one trial is recruiting patients to evaluate the efficacy, safety and tolerability of DHA + PP (3 days) with imported *P. vivax* malaria (NCT02110784).

So far, the use of ACT for non-falciparum malaria, in particular *P. vivax*, has been jeopardized by the supposedly higher economic costs[98]. This judgement is based on assumption rather than evidence and ignores the malariometric advantages of ACT, declining costs and practical, organizational advantages of a unified ACT-based regimen. Studies evaluating the cost-effectiveness of ACT-based strategy for non-falciparum malaria should be conducted.

The use of ACT for non-complicated non-falciparum malaria infection is convenient but may imply the risks’ of a higher utilization of the combination drug, with two possible drawbacks. Firstly, the availability of good quality drugs may be limited in malaria endemic countries[99]; however, there are important caveats to accurately estimate the prevalence and distribution of poor quality anti-malaria drugs. Secondly, inadequate use of ACT (ACT is more expensive than chloroquine) by the population in resource-challenged settings can possibly lead to resistance to both *P. falciparum* and non-falciparum strains[100].

In contrast to *P. falciparum* malaria, *P. vivax*, and to a lesser extent *P. ovale*, form hypnozoites, which remain dormant in liver cells after an acute infection. Therefore, in *P. vivax* infections (and also *P. ovale*) the removal of blood schizonts is not sufficient to clear the infection, as the eradication of hypnozoites (“radical cure”) is required to prevent relapses. This is clinically important and cost-effective particularly in travellers who are not likely to be re-infected.

ACT does not affect the hypnozoites in the liver, but ACT with a long half-live partner drug, such as piperaquine 23–28 days[101], (compared to CQ 1–2 months[102]) was demonstrated to better prevent recurrent parasitaemia after day 28[34, 36, 39]¸ up to day 42[38, 55] and 63[34]. AL which has a short half-life, although less susceptible to the appearance of resistant parasites, is associated with a shorter time to *P. vivax* recurrent parasitaemia. However, neither ACT nor CQ is active against *P. vivax* hypnozoites and thus the treatment must be combined with primaquine, currently the only approved drug able to achieve radical cure. WHO guidelines recommend a course of primaquine for the radical treatment of *P. vivax* of at least 14 days[16]. Although not yet marketed and currently under development, tafenoquine (8-aminoquinoline drug) could lead to a game change in treatment and it may be superior to current treatment due to its radical cure capacity. The proposed indication for tafenoquine is the clearance of the hypnozoite stages of *P. vivax* and *P. ovale*. The assessment of the prophylactic activity and pharmacokinetic profile of oral tafenoquine compared to primaquine for inhibition of liver stage malaria infections demonstrated a much improved efficacy of tafenoquine when compared to primaquine[103]. A phase IIb dose-selection study comparing single-dose tafenoquine 300 mg co-administered with CQ for *P. vivax* malaria relapse prevention was more efficacious than CQ alone, with a similar safety profile[104].

### *Plasmodium ovale*and *Plasmodium malariae*malaria

At present, *P. ovale* and *P. malariae* are among the most neglected tropical diseases, particularly in sub-Saharan Africa. This is illustrated by the fact that only three clinical trials[8, 61, 62] were published on ACT for *P. ovale* and *P. malariae* malaria, compared to a wealth of clinical trials on ACT for *P. falciparum* malaria[105]. More evidence on the efficacy and safety of ACT in the treatment of *P. ovale* and *P. malariae* is essential before further discouraging the current use of CQ for non-falciparum malaria. Today, the limited evidence available supports both the use of CQ as well as ACT (AL) for *P. ovale* and *P. malariae*. It is advisable to treat with ACT if species classification is not 100% certain, because *P. ovale* and *P. malariae* often occur in mixed infections with *P. falciparum*, which is likely to be CQ-resistant[8]. Keeping in mind that beyond *in vivo* testing; *in vitro* and *in silico* methods to assess drug resistance of non-falciparum species remain very much limited up to date. *P. ovale* and *P. malariae* are considered sensitive to CQ. However, there is one report on CQ-resistant *P. malariae* from South Sumatra, Indonesia[17]. Simultaneous use of two anti-malarials of different modes of action (as with ACT) will reduce the chance of selection of a resistant strain[96]. Of concern are the results of a trial conducted in Ghanaian schoolchildren, where individuals with microscopically confirmed asymptomatic malaria were treated with dihydroartemisinin-piperaquine and followed for three weeks[106]. A persistent detection of *P. malariae*, *P. ovale curtisi* and *P. ovale wallikeri* was observed. This apparent “treatment failure” requires more investigation. It is important to note, however, that *in vivo* anti-malarial drug efficacy cannot really be determined from data derived from outcomes of asymptomatic infections. Furthermore, their method of measurement of outcome, PCR, is not yet validated as a primary endpoint method for anti-malarial drug trials. It is speculated by the authors that a relapse of *P. ovale* could explain the persistent detection of parasites; however, the prophylactic effect of piperaquine which is generally assumed to last more than three weeks makes this less plausible[106]. Furthermore, it is possible that only gametocytes of *P. malariae* and *P. ovale* were detected by PCR, and not the asexual stages, as it is known for *P. falciparum* that gametocyte carriage following a dihydroartemisinin-piperaquine regimen is more frequent than for other artemisinin-based combinations (e.g. AL)[107]. In *P. malariae*, the long intra-erythrocytic cycle of three days may explain their persistence due to dihydroartemisinin short T_max_ (±2 h) and rapid clearance by CYP3A4/5 in the liver, and, therefore, may be more likely to survive after each dose of artemisinin. Similar observations have been made concerning delayed parasite clearance times in *P. malariae* in Madagascar, where a patient treated with CQ was still positive for *P. malariae* (detected by PCR) on day 7, although it was subsequently cleared with no late recurrence[108]. Another study, with CQ for *P. malariae*, showed a significant lower clinical response rate, with nearly 20% of patients still parasitaemic by day 2 and one patient remaining parasitaemic on day 4[109]. CQ’s marked stage specificity of action may account for reports of delayed parasite clearance times[109]. Although highly desirable, randomized controlled trials testing different formulations of ACT versus CQ in the treatment of *P. ovale* and *P. malariae* mono-infections are difficult to realize due to patient’s safety issues; misdiagnosis of *P. falciparum* malaria can have fatal consequences.

### *Plasmodium knowlesi*malaria

Although currently randomized clinical trials on the treatment of *P. knowlesi* are lacking, several studies have demonstrated the efficacy and safety of ACT (particularly AL)[65, 66]. Although CQ is efficacious (and cheap) for non-severe *P. knowlesi* malaria, it is recommended, in view of the potential severity and velocity at which the disease can develop, to use an ACT that is suited for the treatment of *P. falciparum* or *P. malariae* for uncomplicated *P. knowlesi* malaria. This is particularly relevant for elderly patients because they are at greater risk of severe disease due to the strong correlation between age and parasite count[46, 110, 111]. The faster parasite clearance properties of AL are more likely to prevent the most common complication of severe knowlesi malaria; hyperparasitaemia[46]. The importance of early diagnosis[112] and rapid treatment is underlined by the fact that parasite biomass is the major independent risk factor for severe knowlesi malaria[46]. Parenteral artesunate, which is associated with short parasite clearance times, is apparently preferable as treatment of severe knowlesi malaria[46] as it is for falciparum malaria.

In studies conducted in Sarawak, it was demonstrated that whilst most patients with microscopy-diagnosed *P. malariae* infection harboured *P. knowlesi*; approximately 10% were actually infected with *P. falciparum*[113]. In travellers returning from Malaysia and other *P. knowlesi* endemic areas, with a high prevalence of CQ-resistant falciparum malaria, negligent use of CQ for misdiagnosed *P. falciparum* malaria may have detrimental consequences. Also, resistance of *P. knowlesi* to anti-malarial drugs have not been observed yet[114]. Moreover, artesunate offers a number of advantages over quinine in terms of not requiring rate-controlled infusion or cardiac monitoring[16]. *Plasmodium knowlesi* was responsible for almost half of the malaria deaths in Sabah (Borneo), due to misdiagnosis with *P. malariae* and delayed administration of artesunate for severe non-falciparum malaria[115]. In a retrospective study, parasite clearance time of AL and injectable artesunate, compared to CQ and quinine, was faster, with fewer deaths with artesunate than quinine[66]. More trials are needed and currently two trials are initiated: “ACT KNOW” (Clinicaltrials.gov: NCT01708876[116]), a RCT to test whether fixed combination of artesunate-mefloquine is superior to CQ and “CAN KNOW” (NCT02001012), a RCT to test whether AL is superior to CQ.

## Conclusions

The considerable burden of non-falciparum malaria (predominantly *P. vivax*) in endemic areas and among travellers, the clear recognition as non-benign infection and the emergence of CQ-resistant strains call for a paradigm change in the way non-falciparum malaria infections are treated. Although non-falciparum malaria remains susceptible to CQ in parts of the world, the role of ACT in the treatment of non-falciparum malaria in endemic as well as non-endemic areas should be acknowledged. ‘Even’ if most non-falciparum infections do not take a potentially life-threatening course, the shorter parasite and fever clearance times alone make a strong case for ACT following establishment of the correct diagnosis. A more uniform treatment for malaria would offer important organizational, logistical and economic advantages, next to malariologic advantages of ACT in preventing resistance, showing rapid parasite and fever clearance times and good cure rates. However, the incomplete nature of current data on the efficacy of ACT in the treatment of *P. ovale*, *P. malariae* and *P. knowlesi* in endemic areas but also as imported conditions compels current malaria researchers to evaluate the efficacy, safety and cost-effectiveness of these drugs in randomized controlled clinical trials. Because most studies have been carried out in endemic settings, one must be careful transferring these results directly to non-immune travellers with imported non-falciparum malaria. On the other hand, European treatment guidelines are mainly based on data from studies carried out in endemic areas, since there is a paucity of original prospective treatment data for non-immune patients[21]. It must be acknowledged that in most settings, there are too few participants for large-scale trials assessing ‘one size fits it all’ strategies for non-falciparum malaria in and outside endemic areas. Therefore, the publication of observational data derived from mouse models, pharmacokinetic data from clinical trials should be encouraged. Studies evaluating the cost-effectiveness of ACT-based strategy for non-falciparum malaria should be conducted[117].

## Electronic supplementary material

Additional file 1:**This documents describes the full methods of the present review.**(DOC 33 KB)

Additional file 2:**This PDF file depicts tables with the exact search strategy.**(PDF 56 KB)

Additional file 3:**This document provides basic background information on** ***P. ovale,*** ***P. malariae*** **and** ***P. knowlesi*** **infections.**(DOC 178 KB)
