# Supplementary material for: Efficacy and safety of artemisinin combination therapy (ACT) for non-falciparum malaria: a systematic review
Source: Malar J. 2014 Nov 26;13:463. doi: 10.1186/1475-2875-13-463 (PMC4258384; doi:10.1186/1475-2875-13-463)
Supplement: Supplementary file 1 — Additional file 1:This documents describes the full methods of the present review.(DOC 33 KB) [file 12936_2014_3620_MOESM1_ESM.doc]

**Additional file 1: Methods section**

A clinical librarian (IMN) searched the electronic databases Ovid Medline (1946 to November 2014), Ovid Embase (1947 to November 2014), Cochrane Central Register of Controlled Trials (November 2014), Web of Science (1975 to November 2014), CINAHL Plus with Full Text (1937 to November 2014), African Index Medicus (1933 to November 2014), African Journals Online (AJOL) (from inception to November 2014), Google Scholar (without patents and citations), Biosis Previews (1993 to November 2014) and PubMed (non-MEDLINE citations) (1947 to November 2014) to identify studies. The search strategy consisted of free-text words and subject headings related to non-falciparum malaria; *P. vivax, P. ovale,* *P. malariae* and *P. knowlesi.*  The search strategy was not limited by study design or language. For *P. malariae, P. ovale* and *P. knowlesi* case-series and case reports were also included. The full search strategy is described in Additional file 2. Because no evidence of studies evaluating ACTs for travellers could be identified, we removed the word “travellers” from the registered PROSPERO title. Unpublished data (e.g. conference or meeting abstracts) were excluded. Bibliographies of relevant studies retrieved from the studies were screened for additional publications. Endnote X7.2 (Thomson Reuters) was used to manage, de-duplicate and screen the references for eligibility. Studies retrieved were eligible for inclusion if they satisfied all selection criteria. The study population consisted of patients with malaria (*P. ovale, P. vivax, P. malariae & P. knowlesi*) of all age groups. Treatment consisted of an artemisinin derivate or artemisinin combination treatment (ACT). Studies evaluating artemisinin mono-therapy were excluded. Only phase III/IV clinical trials were included. Eligibility assessment of studies found was performed independently in an blinded standardized manner by two reviewers (BJV and DK). One author (DK) extracted the following study characteristics: first author, year of publication, study setting, study design, characteristics of trial participants, type & number of controls, antimalarial drug(s) tested and the main outcomes; fever clearance time (FCT), parasite clearance time (PCT) and clinical cure and parasitological failure rates on days 0, 1, 2, 3, 7, 14, 21, 28 42,56 and 63. Table 2 depicts efficacy data and includes only intention-to-treat analysis (α), and if not available, the per-protocol (β) efficacy data . Data was double checked for all articles included in the qualitative synthesis.

**Reference**

1. **Methods for surveillance of antimalarial drug efficacy**. Edited by; World Health Organization, 2009.
