# Supplementary material for: Efficacy and safety of artemisinin combination therapy (ACT) for non-falciparum malaria: a systematic review
Source: Malar J. 2014 Nov 26;13:463. doi: 10.1186/1475-2875-13-463 (PMC4258384; doi:10.1186/1475-2875-13-463)
Supplement: Supplementary file 2 — Additional file 2:This PDF file depicts tables with the exact search strategy.(PDF 56 KB) [file 12936_2014_3620_MOESM2_ESM.pdf]

Database: **Ovid MEDLINE** 1946 to November 2014
Date of search: 16 November 2014

#	Searches	Results
1	Plasmodium vivax/ or Malaria, Vivax/ or Plasmodium ovale/ or Plasmodium malariae/ or Plasmodium knowlesi/ or (vivax or ovale or malariae or knowlesi or non-falciparum or imported malaria or mixed species).ti,ab,kw.	15965
2	Artemisinins/ or (artemis* or artesun* or artemether* or arteflene* or artemotil or arteether or dihydroarte* or quinghaosu or qinghaosu or quinhaosu).ti,ab,kw.	9074
3	1 and 2	417

Database: **Ovid Embase** 1947 to November 2014
Date of search: 16 November 2014

#	Searches	Results
1	*Plasmodium vivax/ or *Plasmodium vivax malaria/ or *Plasmodium ovale/ or *Plasmodium ovale malaria/ or *Plasmodium malariae/ or *Plasmodium malariae infection/ or *Plasmodium knowlesi/ or *Plasmodium knowlesi malaria/ or (vivax or ovale or malariae or knowlesi or non-falciparum or imported malaria or mixed species).ti,ab,kw.	19619
2	artemisinin derivative/ or artemisinin/ or artesunate/ or artemether/ or arteflene/ or arteether/ or dihydroartemisinin/ or (artemis* or artesun* or artemether* or arteflene* or artemotil or arteether or dihydroarte* or quinghaosu or qinghaosu or quinhaosu).ti,ab,kw.	15091
3	1 and 2	775
4	limit 3 to (conference abstract or conference paper or conference proceeding or "conference review")	102
5	3 not 4	673

Database: **Cochrane Central Register of Controlled Trials** (Cochrane Library, November 2014)
Date of Search: 16 November 2014

#	Searches	Results
1	(vivax or ovale or malariae or knowlesi or non-falciparum or "imported malaria" or "mixed species"):ti,ab,kw	495
2	(artemis* or artesun* or artemether* or arteflene* or artemotil or arteether or dihydroarte* or quinghaosu or qinghaosu or quinhaosu):ti,ab,kw	1089
3	#1 and #2 in Trials	72

Database: **Web of Science** 1975 to November 2014
Search date: 16 November 2014

#	Searches	Results
1	TS=(vivax or ovale or malariae or knowlesi or non-falciparum or "imported malaria" or "mixed species")	16519
2	TS=(artemis* or artesun* or artemether* or arteflene* or artemotil or arteether or dihydroarte* or quinghaosu or qinghaosu or quinhaosu)	15171
3	#1 AND #2	448

Database: **CINAHL Plus with Full Text** 1937 to November 2014
Search date: 16 November 2014

#	Searches	Results
S1	TX (vivax or ovale or malariae or knowlesi or non-falciparum or "imported malaria" or "mixed species")	2405
S2	TX (artemis* or artesun* or artemether* or arteflene* or artemotil or arteether or dihydroarte* or quinghaosu or qinghaosu or quinhaosu)	1555
S3	S1 AND S2	64

Database: **African Index Medicus** 1993 to November 2014
Date of search: 16 November 2014

#	Searches	Results
1	vivax OR ovale OR malariae OR knowlesi OR non-falciparum	11

Database: **African Journals Online (AJOL)** from inception to November 2014
Date of search: 16 November 2014

#	Searches	Results
1	(vivax or ovale or malariae or knowlesi or non-falciparum or "imported malaria" or "mixed species") AND (artemis* or artesun* or artemether* or arteflene* or artemotil or arteether or dihydroarte* or quinghaosu or qinghaosu or quinhaosu)	2

Database: **Google Scholar** (without patents and citations)
Date of search: 16 November 2014

#	Searches	Results
1	allintitle:vivax | ovale | malariae | knowlesi | non-falciparum | "imported malaria" | "mixed species" allintitle:artemisinin | artesunate | artemether | arteflene | artemotil | arteether | dihydroartemisinin | quinghaosu | qinghaosu | quinhaosu	51

Database: **LILACS (Latin-American and Caribbean Health Sciences Literature)** 1982 to Nov 2014
Date of search: 16 November 2014

#	Searches	Results
1	TW:(vivax or ovale or malariae or knowlesi or non-falciparum) AND TW:(artemis$ or artesun$ or artemether$ or arteflene$ or artemotil or arteether or dihydroarte$ or quinghaosu or qinghaosu or quinhaosu)	6

Database: **BIOSIS Previews** 1993 to November 2014
Date of search: 16 November 2014

#	Searches	Results
1	(vivax or ovale or malariae or knowlesi or non-falciparum or imported malaria or mixed species).mp.	9420
2	(artemis* or artesun* or artemether* or arteflene* or artemotil or arteether or dihydroarte* or quinghaosu or qinghaosu or quinhaosu).mp.	13296
3	1 and 2	229

Database: **PubMed (non-MEDLINE citations)** 1947 to November 2014
Date of search: 16 November 2014

#	Searches	Results
1	((vivax[tiab] OR ovale[tiab] OR malariae[tiab] OR knowlesi[tiab] OR non-falciparum[tiab] OR imported malaria[tiab] OR mixed species[tiab]) AND (artemis*[tiab] OR artesun*[tiab] OR artemether*[tiab] OR arteflene*[tiab] OR artemotil[tiab] OR arteether[tiab] OR dihydroarte*[tiab] OR quinghaosu[tiab] OR qinghaosu[tiab] OR quinhaosu[tiab])) NOT medline[sb]	49
