# Supplementary material for: Efficacy and safety of artemisinin combination therapy (ACT) for non-falciparum malaria: a systematic review
Source: Malar J. 2014 Nov 26;13:463. doi: 10.1186/1475-2875-13-463 (PMC4258384; doi:10.1186/1475-2875-13-463)
Supplement: Supplementary file 3 — Additional file 3:This document provides basic background information on P. ovale, P. malariae and P. knowlesi infections.(DOC 178 KB) [file 12936_2014_3620_MOESM3_ESM.doc]

**Supplementary file III: Supporting information *P. ovale, P. malariae* and *P. knowlesi.***

***P. ovale*  and *P. malariae* malaria**

*Plasmodium ovale* and *P. malariae* are found where malaria is endemic, causing an estimated 60 million clinical cases each year . Despite the fact that they only account for a small proportion of all malaria cases (<15%), their prevalence is likely to be underestimated, because they are frequently missed on routine microscopic examination . Infections due to ovale and malariae malaria are mild with low parasitemias (<1000/ μl), with fever classically every 3 days (quartan malaria, *P. malariae)* and every 2 days (tertian malaria, *P. ovale*). *P. ovale* actually consists of two subspecies that co-circulate in Africa and Asia and that are unable to recombine genetically; *P. ovale curtisi* and *P. ovale wallikeri* . Limited information is available on differences in clinical symptoms, relapse profile, or accuracy of RDT results between these *P. ovale* species. Severe manifestations of both types (*P. ovale* and *P. malariae)* are relatively rare. Chronic infections can cause anaemia and in the case of *P. malariae* an immune complex-mediated nephrotic syndrome. Theoretically, *P. ovale* hypnozoites can cause relapsing disease, which has implications for treatment. These hypnozoites however have never been demonstrated by biological experiments, and the few reports published on relapses have conflicting results. *P. malariae* can cause prolonged subpatent parasitemia and may reappear 20 years after living in an endemic area .

***P. knowlesi* malaria**

Discovered by Franchini in 1927 in its natural host, the long-tailed macaque (*Macaca fascicularis)*, the simian parasite *Plasmodium knowlesi* was found to be transmissible to humans by Knowles and Das Gupta in 1932 . Currently, it is an important cause of malaria in Malaysian Borneo and Peninsular and should be considered in ill travellers returning from this region . The first naturally acquired infection was reported in 1965 in an American traveller returning from Malaysia . Reports of human infection were scarce until 2004, when infection with *P. knowlesi* was described among 120 patients in Malaysia . This study showed that *P. knowlesi* accounted for over half of all malaria cases in the Kapit division of the Malaysian state of Sarawak (Borneo). Polymerase chain reaction analysis (PCR) of archival blood films from patients earlier diagnosed with *P. malariae* infections from Borneo confirmed that infections with *P. knowlesi* already occurred in 1996 . These discoveries led to the recognition of *P. knowlesi*  as the fifth human malaria parasite . Recent estimations show a ten-fold increase of *P. malariae/P. knowlesi* notifications between 2004 (n = 59) and 2011 (n = 703) in Sabah (Borneo), following reduced transmission of the other human *Plasmodium* species . *P. knowlesi* was not earlier recognized by microscopy, because of its morphological similarities with *P. malariae* . It has also been reported in other parts of forested regions in South and East Asia . *P. knowlesi* is transmitted by mosquitoes of the *Anopheles leucosphyrus* group . Clinical trials evaluating antimalarial drugs have only been conducted in Borneo, with case reports or series from travellers returning from Thailand , Burma , Vietnam , Cambodia , Singapore , Philippines , Indonesia and other parts of the world . *P. knowlesi* malaria is not “benign” malaria but a potentially life-threatening disease instead. It can cause severe symptoms including a high parasite density (75,000 to 765,000 parasites/µL) (due to its 24-hour replication cycle), hypotension, hypoglycaemia, respiratory distress syndrome and hepatic and renal dysfunction . In a prospective comparative study in Malaysia, *P. knowlesi* was associated with a 3-fold (95%CI 1.19–7.38) greater risk of severity than *P. falciparum* (P=0.02) . Diagnosis is made by microscopic examination, however this is challenging due to its resemblance with *P. falciparum* in the early trophozoite stage and with *P. malariae* in the late and mature trophozoites, gametocytes, and schizonts . For well-equipped settings, real-time PCR has been developed to diagnose *P. knowlesi* . A faster technique utilizing simpler and cheaper methodology is loop-mediated isothermal amplification (LAMP). It targets *P. knowlesi*-specific primers against β-tubulin and apical membrane antigen-1 . Rapid diagnostic tests (RDTs), based on Plasmodium lactate dehydrogenase (pan-pLDH) and genus-specific aldolase (pan-aldolase) cannot differentiate between non-falciparum species . Recently, a model using different anti-pLDH antibody combinations was developed, with which *P. knowlesi* could be distinguished from the other human Plasmodium species . For further reading, see the detailed overview of human *P. knowlesi* infection review of Singh and Daneshvar .

**References**

1. Mueller I, Zimmerman PA, Reeder JC: **Plasmodium malariae and Plasmodium ovale--the "bashful" malaria parasites**. *Trends Parasitol* 2007, **23**:278-283.

2. May J, Mockenhaupt FP, Ademowo OG, et al.: **High rate of mixed and subpatent malarial infections in southwest Nigeria**. *Am J Trop Med Hyg* 1999, **61**:339-343.

3. Mehlotra RK, Lorry K, Kastens W, et al.: **Random distribution of mixed species malaria infections in Papua New Guinea**. *Am J Trop Med Hyg* 2000, **62**:225-231.

4. Collins WE, Jeffery GM: **Plasmodium malariae: parasite and disease**. *Clin Microbiol Rev* 2007, **20**:579-592.

5. Mayxay M, Pukrittayakamee S, Newton PN, et al.: **Mixed-species malaria infections in humans**. *Trends in Parasitology* 2004, **20**:233-240.

6. Snounou G, Pinheiro L, Goncalves A, et al.: **The importance of sensitive detection of malaria parasites in the human and insect hosts in epidemiological studies, as shown by the analysis of field samples from Guinea Bissau**. *Trans R Soc Trop Med Hyg* 1993, **87**:649-653.

7. Sutherland CJ, Tanomsing N, Nolder D, et al.: **Two nonrecombining sympatric forms of the human malaria parasite Plasmodium ovale occur globally**. *J Infect Dis* 2010, **201**:1544-1550.

8. Richter J, Franken G, Mehlhorn H, et al.: **What is the evidence for the existence of Plasmodium ovale hypnozoites?** *Parasitol Res* 2010, **107**:1285-1290.

9. Siala E, Khalfaoui M, Bouratbine A, et al.: **[Relapse of Plasmodium malariae malaria 20 years after living in an endemic area]**. *Presse Med* 2005, **34**:371-372.

10. Knowles R, Das Gupta B: **A study of monkey–malaria and its experimental transmission to man.** *Ind Med Gaz.* 1932, **67**:301–320.

11. Muller M, Schlagenhauf P: **Plasmodium knowlesi in travellers, update 2013**. *Int J Infect Dis* 2014.

12. Chin W, Contacos PG, Coatney GR, et al.: **A NATURALLY ACQUITED QUOTIDIAN-TYPE MALARIA IN MAN TRANSFERABLE TO MONKEYS**. *Science* 1965, **149**:865.

13. Singh B, Kim Sung L, Matusop A, et al.: **A large focus of naturally acquired Plasmodium knowlesi infections in human beings**. *Lancet* 2004, **363**:1017-1024.

14. Lee KS, Cox-Singh J, Singh B: **Morphological features and differential counts of Plasmodium knowlesi parasites in naturally acquired human infections**. *Malar J* 2009, **8**:73.

15. White NJ: **Plasmodium knowlesi: the fifth human malaria parasite**. *Clin Infect Dis* 2008, **46**:172-173.

16. William T, Rahman HA, Jelip J, et al.: **Increasing Incidence of Plasmodium knowlesi Malaria following Control of P. falciparum and P. vivax Malaria in Sabah, Malaysia**. *Plos Neglected Tropical Diseases* 2013, **7**:9.

17. Barber BE, William T, Grigg MJ, et al.: **Limitations of microscopy to differentiate Plasmodium species in a region co-endemic for Plasmodium falciparum, Plasmodium vivax and Plasmodium knowlesi**. *Malaria Journal* 2013, **12**:6.

18. Baird JK: **Malaria zoonoses**. *Travel Med Infect Dis* 2009, **7**:269-277.

19. Collins WE: **Plasmodium knowlesi: a malaria parasite of monkeys and humans**. *Annu Rev Entomol* 2012, **57**:107-121.

20. Putaporntip C, Hongsrimuang T, Seethamchai S, et al.: **Differential prevalence of Plasmodium infections and cryptic Plasmodium knowlesi malaria in humans in Thailand**. *J Infect Dis* 2009, **199**:1143-1150.

21. Jongwutiwes S, Putaporntip C, Iwasaki T, et al.: **Naturally acquired Plasmodium knowlesi malaria in human, Thailand**. *Emerg Infect Dis* 2004, **10**:2211-2213.

22. Orth H, Jensen BO, Holtfreter MC, et al.: **Plasmodium knowlesi infection imported to Germany, January 2013**. *Euro Surveillance: Bulletin Europeen sur les Maladies Transmissibles = European Communicable Disease Bulletin* 2013, **18**.

23. Zhu HM, Li J, Zheng H: **[Human natural infection of Plasmodium knowlesi]**. *Zhongguo Ji Sheng Chong Xue Yu Ji Sheng Chong Bing Za Zhi* 2006, **24**:70-71.

24. Jiang N, Chang Q, Sun X, et al.: **Co-infections with Plasmodium knowlesi and other malaria parasites, Myanmar**. *Emerg Infect Dis* 2010, **16**:1476-1478.

25. Van den Eede P, Van HN, Van Overmeir C, et al.: **Human Plasmodium knowlesi infections in young children in central Vietnam**. *Malar J* 2009, **8**:249.

26. Marchand RP, Culleton R, Maeno Y, et al.: **Co-infections of Plasmodium knowlesi, P. falciparum, and P. vivax among Humans and Anopheles dirus Mosquitoes, Southern Vietnam**. *Emerg Infect Dis* 2011, **17**:1232-1239.

27. Khim N, Siv S, Kim S, et al.: **Plasmodium knowlesi infection in humans, Cambodia, 2007-2010**. *Emerg Infect Dis* 2011, **17**:1900-1902.

28. Ng OT, Ooi EE, Lee CC, et al.: **Naturally acquired human Plasmodium knowlesi infection, Singapore**. *Emerg Infect Dis* 2008, **14**:814-816.

29. Jeslyn WP, Huat TC, Vernon L, et al.: **Molecular epidemiological investigation of Plasmodium knowlesi in humans and macaques in Singapore**. *Vector Borne Zoonotic Dis* 2011, **11**:131-135.

30. Luchavez J, Espino F, Curameng P, et al.: **Human Infections with Plasmodium knowlesi, the Philippines**. *Emerg Infect Dis* 2008, **14**:811-813.

31. **Simian malaria in a U.S. traveler--New York, 2008**. *MMWR Morb Mortal Wkly Rep* 2009, **58**:229-232.

32. Dacuma MGB, Hallett R, Dimalibot J, et al.: **Epidemiology of Plasmodium Falciparum, P. Vivax, and Zoonotic P. Knowlesi in Southern Mindanao, the Philippines**. *American Journal of Tropical Medicine & Hygiene* 2010, **83**:170.

33. Figtree M, Lee R, Bain L, et al.: **Plasmodium knowlesi in human, Indonesian Borneo**. *Emerg Infect Dis* 2010, **16**:672-674.

34. Kantele A, Marti H, Felger I, et al.: **Monkey malaria in a European traveler returning from Malaysia**. *Emerg Infect Dis* 2008, **14**:1434-1436.

35. Hoosen A, Shaw MT: **Plasmodium knowlesi in a traveller returning to New Zealand**. *Travel Medicine & Infectious Disease* 2011, **9**:144-148.

36. Berry A, Iriart X, Wilhelm N, et al.: **Imported Plasmodium knowlesi malaria in a French tourist returning from Thailand**. *Am J Trop Med Hyg* 2011, **84**:535-538.

37. Link L, Bart A, Verhaar N, et al.: **Molecular detection of Plasmodium knowlesi in a Dutch traveler by real-time PCR**. *J Clin Microbiol* 2012, **50**:2523-2524.

38. Ehrhardt J, Trein A, Kremsner PG, et al.: **Plasmodium knowlesi and HIV co-infection in a German traveller to Thailand**. *Malar J* 2013, **12**:283.

39. Ta TT, Salas A, Ali-Tammam M, et al.: **First case of detection of Plasmodium knowlesi in Spain by Real Time PCR in a traveller from Southeast Asia**. *Malar J* 2010, **9**:219.

40. van Hellemond JJ, Rutten M, Koelewijn R, et al.: **Human Plasmodium knowlesi infection detected by rapid diagnostic tests for malaria**. *Emerg Infect Dis* 2009, **15**:1478-1480.

41. Bronner U, Divis PC, Farnert A, et al.: **Swedish traveller with Plasmodium knowlesi malaria after visiting Malaysian Borneo**. *Malar J* 2009, **8**:15.

42. Cox-Singh J, Davis TM, Lee KS, et al.: **Plasmodium knowlesi malaria in humans is widely distributed and potentially life threatening**. *Clin Infect Dis* 2008, **46**:165-171.

43. Barber BE, William T, Grigg MJ, et al.: **A prospective comparative study of knowlesi, falciparum, and vivax malaria in Sabah, Malaysia: high proportion with severe disease from Plasmodium knowlesi and Plasmodium vivax but no mortality with early referral and artesunate therapy**. *Clinical Infectious Diseases* 2013, **56**:383-397.

44. Lucchi NW, Poorak M, Oberstaller J, et al.: **A new single-step PCR assay for the detection of the zoonotic malaria parasite Plasmodium knowlesi**. *PLoS One* 2012, **7**:e31848.

45. Iseki H, Kawai S, Takahashi N, et al.: **Evaluation of a loop-mediated isothermal amplification method as a tool for diagnosis of infection by the zoonotic simian malaria parasite Plasmodium knowlesi**. *J Clin Microbiol* 2010, **48**:2509-2514.

46. Lau YL, Fong MY, Mahmud R, et al.: **Specific, sensitive and rapid detection of human plasmodium knowlesi infection by loop-mediated isothermal amplification (LAMP) in blood samples**. *Malar J* 2011, **10**:197.

47. Barber BE, William T, Grigg MJ, et al.: **Evaluation of the Sensitivity of a pLDH-Based and an Aldolase-Based Rapid Diagnostic Test for Diagnosis of Uncomplicated and Severe Malaria Caused by PCR-Confirmed Plasmodium knowlesi, Plasmodium falciparum, and Plasmodium vivax**. *Journal of Clinical Microbiology* 2013, **51**:1118-1123.

48. Piper RC, Buchanan I, Choi YH, et al.: **Opportunities for improving pLDH-based malaria diagnostic tests**. *Malar J* 2011, **10**:213.

49. Singh B, Daneshvar C: **Human infections and detection of Plasmodium knowlesi**. *Clin Microbiol Rev* 2013, **26**:165-184.
